# Genome-wide analysis and identification of the TBL gene family in *Eucalyptus grandis*

**DOI:** 10.3389/fpls.2024.1401298

**Published:** 2024-08-07

**Authors:** Jiye Tang, Tenghong Ling, Huiling Li, Chunjie Fan

**Affiliations:** ^1^ Guangdong Key Laboratory for Innovative Development and Utilization of Forest Plant Germplasm, College of Forestry and Landscape Architectures, South China Agricultural University, Guangzhou, China; ^2^ State Key Laboratory of Tree Genetics and Breeding, Key Laboratory of State Forestry and Grassland Administration on Tropical Forestry, Research Institute of Tropical Forestry, Chinese Academy of Forestry, Guangzhou, China

**Keywords:** TBL, *Eucalyptus grandis*, stress resistance, xylan acetylation, gene family

## Abstract

The *TRICHOME BIREFRINGENCE-LIKE* (*TBL*) gene encodes a class of proteins related to xylan acetylation, which has been shown to play an important role in plant response to environmental stresses. This gene family has been meticulously investigated in *Arabidopsis thaliana*, whereas there have been no related reports in *Eucalyptus grandis*. In this study, we identified 49 TBL genes in *E. grandis*. A conserved amino acid motif was identified, which plays an important role in the execution of the function of TBL gene family members. The expression of TBL genes was generally upregulated in jasmonic acid-treated experiments, whereas it has been found that jasmonic acid activates the expression of genes involved in the defense functions of the plant body, suggesting that TBL genes play an important function in the response of the plant to stress. The principle of the action of TBL genes is supported by the finding that the xylan acetylation process increases the rigidity of the cell wall of the plant body and thus improves the plant’s resistance to stress. The results of this study provide new information about the TBL gene family in *E. grandis* and will help in the study of the evolution, inheritance, and function of TBL genes in *E. grandis*, while confirming their functions.

## 1 Introduction

The plant cell wall consists of three main components: cellulose, lignin, and hemicellulose. The wall of a plant cell gives it cellular strength, while functioning as an osmotic barrier. Cell wall structure is important for plant resistance to abiotic stresses and is essential in stress sensing and signaling (Seifert and Blaukopf, 2010). Hemicellulose, a highly branched polysaccharide composed of xylose, galactose, and glucose, which makes up approximately 1/3 of the dry mass of the cell wall (Pauly and Keegstra, 2010), plays an important role in the structure and function of plant cell walls. Among them, xylan is the main hemicellulose in secondary walls of dicotyledons and monocotyledons. O-acetylation is a prevalent substituent in the hemicellulose and also a common method of modification of xylan (Gille and Pauly, 2012; Pawar et al., 2013; Pauly and Ramírez, 2018).

It has been shown that the acetylation of xylan is a cascade-regulated signaling process, with the initial acetyl group coming from CoA (which is the only source of xylan acetyl groups) (Zhong et al., 2020). The first step in the acetylation of the xylan backbone occurs at the Golgi, where RWA proteins are responsible for transferring CoA to the Golgi (Manabe et al., 2011), with the intermediate product AXY9 acting as a bridge to carry the acetyl group first (Schultink et al., 2015); finally, this acetyl group will be transferred ultimately to the xylan backbone in the presence of TBLs (Xiong et al., 2013; Urbanowicz et al., 2014). These studies suggest that TBL gene family proteins function on the xylan acetylation process in the cell wall.

The enzyme encoded by the TBL family of genes played an important role in the O-acetylation of cell wall polymers in *Arabidopsis thaliana* (Yuan et al., 2016a; Zhong et al., 2017; Stranne et al., 2018), which contains the conserved motifs Gly-Asp-Ser, Asp-x-x-His, and TBL, and DUF231 structural domains in the TBL proteins (Bischoff et al., 2010; Gille and Pauly, 2012). It was also found that xylan acetylation was reduced by 7%–20% in Arabidopsis double mutants of TBL genes, such as *tbl3*/*tbl31*, *tbl32*/*tbl33*, and *tbl34*/*tbl35* (Yuan et al., 2016a), whereas in the *tbl29*/*esk1* and *tbl11*/*tbl2* double mutants, xylan acetylation was even significantly reduced by 40% and 55%, respectively (Gao et al., 2017). O-acetylation is prevalent in hardwood xylan, with the degree of acetylation sometimes being as high as 70% (at the C2/C3 position) (Pawar et al., 2013). The degree of acetylation of xylan can regulate the binding of lignin to xylan, thereby affecting biomass energy conversion. At the same time, the degree of acetylation of this xylan also determines its hydrophobicity, which, in turn, affects the structures of the cell wall (Busse-Wicher et al., 2014; Johnson et al., 2017). In poplar, it was found that 63% of xylan is acetylated (Zhong et al., 2018); this suggests that the acetylation of xylan is an important physiological process.

*Eucalyptus* is a tall tree species of the *Myrtaceae* family, native to Australia, which is now widely introduced and planted globally (Trueman et al., 2018). On the one hand, eucalyptus has ideal wood properties suitable for papermaking and hardwood processing, while the essential oils in the tree have an important role in the biomedical field, so it has a great value for utilization (Silva et al., 2003). On the other hand, its high growth capacity and resistance to adversity make it widely cultivated in many countries and generate high economic value (Nogueira et al., 2020). Studies have shown that acetylation of cell wall xylan promotes interactions between cell wall polymers and contributes to increased cell wall rigidity. The increase in cell wall rigidity is important for the normal functioning of the plant body in the process of resistance to adversity and other different biological functions (Pauly et al., 2001; Gille and Pauly, 2012; Bacete et al., 2018). Members of the TBL gene family play an important role in the process of xylan acetylation. Owing to the increased demand in the future (Arya et al., 2009), TBL family genes as a breakthrough to enhance the resilience of eucalyptus are a feasible solution to increase their economic value and meet the market demand. Our study will focus on the TBL gene family of eucalyptus, with the aim of contributing to the study of timber utilization in eucalyptus trees.

## 2 Materials and methods

### 2.1 Collection of materials

Genomic data of *E. grandis* were obtained from Phytozome’s database (Myburg et al., 2014), which includes genome sequence (assembled to chromosome level) and gene structure annotation information. Gene expression data were obtained from the literature (https://onlinelibrary.wiley.com/doi/10.1111/pce.14814) (Fan et al., 2024). Sequence files of TBL-related genes of *A. thaliana* were obtained from the TAIR (https://www.arabidopsis.org/) (Lamesch et al., 2012; Berardini et al., 2015). Genome sequences of *Selaginella moellendorffii*, *Physcomitrium patens*, and *Chlamydomonas reinhardtii* were obtained from the NCBI database (https://www.ncbi.nlm.nih.gov/genome/?term=) (Merchant et al., 2007; Banks et al., 2011; Lang et al., 2018). Genomic data of *Populus alba* were obtained from the research results of the Chinese Academy of Forestry (https://doi.org/10.1007/s11427–018-9455–2) (Liu et al., 2019).

### 2.2 Identification and characterization of TBL family members in *E. grandis*

The genome files were converted into protein sequence files using TBtools (Chen et al., 2020), then the protein sequence data of Arabidopsis and Eucalyptus TBL genes were submitted via the “Blast Compare Two Seqs” plug-in of TBtools. Among the above comparison result files, the TBL gene with the highest match was selected, its ID was copied, and it was submitted to the “Fasta Extract” function plug-in of TBtools along with the TBL protein sequence file, which finally outputted the protein sequence file of the eucalyptus TBL gene. Then, the obtained data were further compared and some sequences with obvious mistakes were deleted.

### 2.3 Protein property analysis

The protein sequence information of the screened TBL gene family members was submitted to TBtools’ “Protein Parameter Calc” program for analysis to obtain tabular data on the physicochemical properties including isoelectric point (pI), hydrophilicity, and molecular mass of the proteins. This study also utilized tools borrowed to predict the subcellular localization of TBL proteins.

### 2.4 Classification and sequence analysis of the TBL genes

The construction of the phylogenetic tree used “one step build ML tree” (i.e., maximum likelihood method to build the phylogenetic tree) of TBtools, the processed TBL protein sequence of eucalyptus was submitted, and “Bootstrap=1000” was set to run the program to get the single-species phylogenetic tree. At the same time, the amino acid sequences of TBL proteins of *E. grandis*, *S. moellendorffii*, and *P. patens* were united to draw a big phylogenetic tree (Subramanian et al., 2019).

### 2.5 Gene structure and conserved motif analysis

After screening the amino acid sequence information of the TBL family proteins of *E. grandis*, the data were submitted to the MEME website (https://memesuite.org/meme/tools/meme) for conserved motif analysis, and the number of conserved motifs was set to 10. Previously predicted sequences that lacked a large number of conserved motifs were considered as pseudogenes and were deleted (Bailey and Elkan, 1994; Bailey et al., 2009). Meanwhile, the gene annotation file of *E. grandis* was downloaded from Phytozome’s database, and the conserved structure domain and gene structure of TBL genes were predicted. Finally, the conserved structure domain prediction file, the gene structure annotation information file, and the phylogenetic tree file were submitted to the “Gene Structure View” visualization plug-in of TBtools for visualization.

### 2.6 Cis-acting element analysis

For cis-acting element analysis, all upstream 2,000 bp of Eucalyptus TBL genes were submitted to the Plant CARE website (https://bioinformatics.psb.ugent.be/webtools/plantcare/html/) (Rombauts et al., 1999; Lescot et al., 2002). The functions related to each cis-acting element are described in the Results section and **Supplementary Table 1**.

### 2.7 Chromosome location and collinearity analysis

Chromosomal localization analysis was performed using the plug-in “Gene Location Visualize from GTF/GFF” on TBtools. Collinearity analysis was performed using the TBtools program “One Step MCScanX-Diamond”. “Dual Systeny Plot” was used for visualization, and the TBL gene ID of *E. grandis* was submitted to be highlighted in the collinearity plot. Finally, the collinearity maps of *E. grandis*–*P. alba* and *E. grandis*–*A. thaliana* were plotted. During the collinearity analysis, the number of CPU for BlastP is 2, the e-value value is 1e−3, and the number of BlastHits is 10.

### 2.8 Expression pattern analysis of TBLs in various tissues and under different stresses

The sample of the expression data measurement comes from clone eucalyptus plant GL1, and the eucalyptus is cultivated in the greenhouse at the Research Institute of Tropical Forestry (113.385°E, 23.191°N), Chinese Academy of Forestry, Guangzhou, China. In terms of hormone-induced and coercion, we determined the expression data of salicylic acid (SA), jasmonic acid (JA), and salt stress. The treatment samples were selected from 3-month-old trees and the height of the plant was 25–35 cm. Hormone treatments involved spraying 100 μM SA and JA onto the foliage, and salt stress treatments entailed spraying the leaves using 200 mM NaCl solution; the leaves were collected after 0, 1, 6, 24, and 168 h of treatment. After the training period is over, gently wipe the root, immediately freeze it in liquid nitrogen, and store it under −80°C. The data of the young leaf, adult leaf, xylem, phloem, and stem apex come from the semi-annual eucalyptus in the greenhouse of the Research Institute of Tropical Forestry. The samples of the 1st (Apex), 3rd, 5th, 7th, 9th, and 11th internodes also come from the above semi-annual big eucalyptus seedlings. Three single plants are gathered to represent a biological repetition, and there are three biological replicates.

For expression mapping, we take the average of three replicates, and for the overall viewing effect, we convert the data to *Y* = Log_2_^(^ *^X^* ^+1)^ (*Y* is the input visualization data and *X* is the original data); i.e., we perform global normalization, so that small fluctuations in the data can be clearly discerned after processing, so that the color contrast of the expression gap embodied in the heatmap after visualization is larger and is more conducive to the observation of the changes in gene expression. Finally, an expression matrix was constructed with time as the horizontal column and gene IDs as the vertical column. Submit the expression matrix to the heat map visualization tool “Heatmap” in TBtools and get the heat map.

In this study, the significance of expression was analyzed and bar graphs were plotted for the *EgTBL36* gene. Significance analysis was done through SPSS software using one-way ANOVA test comparing means, and plotting was done using Origin software (Edwards, 2002).

## 3 Results

### 3.1 Identification and characterization of TBL family members in *E. grandis*

A total of 49 TBL gene family members were identified in *E. grandis* (sequence information for these members can be found in the Appendix). They were renamed according to the nomenclature of their homologs in *A. thaliana* and named *EgTBL1*–*EgTBL49*. The most characteristic sequences of the TBL gene family are the CDS (Cys-Asp-Ser) conserved motifs, which are highly conserved among the 49 TBL family members. The isoelectric points (pI) of the 49 member proteins ranged from 5.37 to 9.64, among which the proteins with pI greater than 7, which are regarded as alkaline proteins, accounted for most of them. Instability index ranged from 30.05 to 61.91. The lengths ranged from 341 to 520 amino acids while the relative molecular masses ranged from 38.41 to 58.98 KD. The aliphatic index was between 62.87 and 86.5, and the grand average of hydropathy was between −0.592 and −0.122, all of which were hydrophilic proteins. Predictions of subcellular localization indicate that most of the TBL family proteins are localized in chloroplasts and vesicles (**Table 1**).

**Table 1:** Physicochemical properties of TBL gene family members.

Sequence ID	Number of amino acids (aa)	Molecular weight (Da)	Theoretical (pI)	Instability index	Aliphatic index	Grand average of hydropathicity	Locus of TBL genes
EgTBL1	520	58,978.74	9.17	61.91	62.87	−0.577	Chloroplast
EgTBL2	411	47,859.7	8.24	58.92	77.81	−0.37	Chloroplast
EgTBL3	415	47,897.36	7.65	52.48	81.52	−0.367	Vacuole
EgTBL4	428	49,222.51	6.68	46	77.22	−0.479	Plasma membrane
EgTBL5	480	54,431.56	8.94	45.64	66.19	−0.582	Mitochondria
EgTBL6	416	47,958.52	9.43	40.63	68.39	−0.509	Vacuole
EgTBL7	411	47,169.02	8.68	47.25	86.5	−0.122	Mitochondria
EgTBL8	418	48,478.82	8.72	58.51	71.15	−0.559	Mitochondria
EgTBL9	409	47,282.42	8.05	41.34	71.49	−0.534	Chloroplast
EgTBL10	446	51,243.01	7	42.86	73.36	−0.426	E.R.
EgTBL11	449	51,141.21	8.74	47.94	77.24	−0.364	Chloroplast
EgTBL12	437	50,422.28	8.33	46.28	69.11	−0.486	Chloroplast
EgTBL13	483	53,244.82	9.42	44.31	74.62	−0.331	E.R.
EgTBL14	419	47,973.08	9.34	40.05	75.39	−0.421	Peroxisomes
EgTBL15	459	51,671.69	8.91	48.83	73.92	−0.43	Vacuole
EgTBL16	437	50,310.87	5.94	50.16	78.1	−0.475	Vacuole
EgTBL17	392	44,388.1	6.19	36.62	66.17	−0.483	Nucleus
EgTBL18	422	47,414.36	8.88	46.59	76.82	−0.298	Vacuole
EgTBL19	435	49,074.53	6.57	40.94	71.56	−0.442	Chloroplast
EgTBL20	444	51,010.3	8.58	40.17	72.52	−0.33	Cytoplasm
EgTBL21	381	43,149.68	6.03	37.3	67.61	−0.479	Cytoplasm
EgTBL22	443	50,912.11	8.26	42.66	72.69	−0.321	Cytoplasm
EgTBL23	455	51,036.87	5.86	49.9	73.74	−0.34	Chloroplast
EgTBL24	431	49,097.5	6.18	44.09	77.61	−0.479	Vacuole
EgTBL25	446	51,143.94	5.53	44.36	78.03	−0.393	Vacuole
EgTBL26	411	47,148.46	9.64	35.13	72.53	−0.234	Chloroplast
EgTBL27	452	50,674.36	5.61	47.31	75.75	−0.339	Chloroplast
EgTBL28	484	56,107.68	6.18	56.07	69.44	−0.592	Nucleus
EgTBL29	362	41,400.37	9.23	39.53	79.39	−0.286	E.R.
EgTBL30	432	49,493.71	7.18	41.73	77.11	−0.397	Chloroplast
EgTBL31	480	56,192.44	9.13	50.59	80.19	−0.524	Chloroplast
EgTBL32	483	54,074.08	6.61	58.05	68.39	−0.466	Vacuole
EgTBL33	423	48,423.74	8.85	40.29	73.29	−0.312	Vacuole
EgTBL34	382	44,367.82	9.13	51.3	75.31	−0.477	Vacuole
EgTBL35	365	41,045.69	7.03	37.48	79.59	−0.187	Extracellular
EgTBL36	387	45,577.98	8.61	56.63	70.98	−0.556	Nucleus
EgTBL37	373	42,251.24	9.01	40.16	74.24	−0.308	Chloroplast
EgTBL38	364	40,644	6.14	38.18	77.64	−0.302	Vacuole
EgTBL39	365	42,622.89	9.39	38.74	72.11	−0.338	Nucleus
EgTBL40	365	41,807.56	8.98	33.28	79.34	−0.326	Vacuole
EgTBL41	341	38,409.98	8.54	41.66	75.45	−0.307	Cytoplasm
EgTBL42	357	39,912.22	5.37	39.27	79.75	−0.278	Golgi apparatus
EgTBL43	365	41,211.97	8.67	34.2	79.81	−0.223	Extracellular
EgTBL44	397	45,276.43	6.07	30.05	79.37	−0.332	Chloroplast
EgTBL45	390	44,085.36	8.87	30.19	73.49	−0.286	Chloroplast
EgTBL46	460	52,886.78	8.89	50.49	81.37	−0.297	Vacuole
EgTBL47	426	49,029.36	8.22	42.18	75.7	−0.315	Vacuole
EgTBL48	442	51,567.69	8.57	50.8	76.06	−0.561	Nucleus
EgTBL49	411	47,589.3	8.61	48.82	77.83	−0.436	Mitochondria

### 3.2 Phylogenetic tree and sequence structure analysis

**Figure 1:**
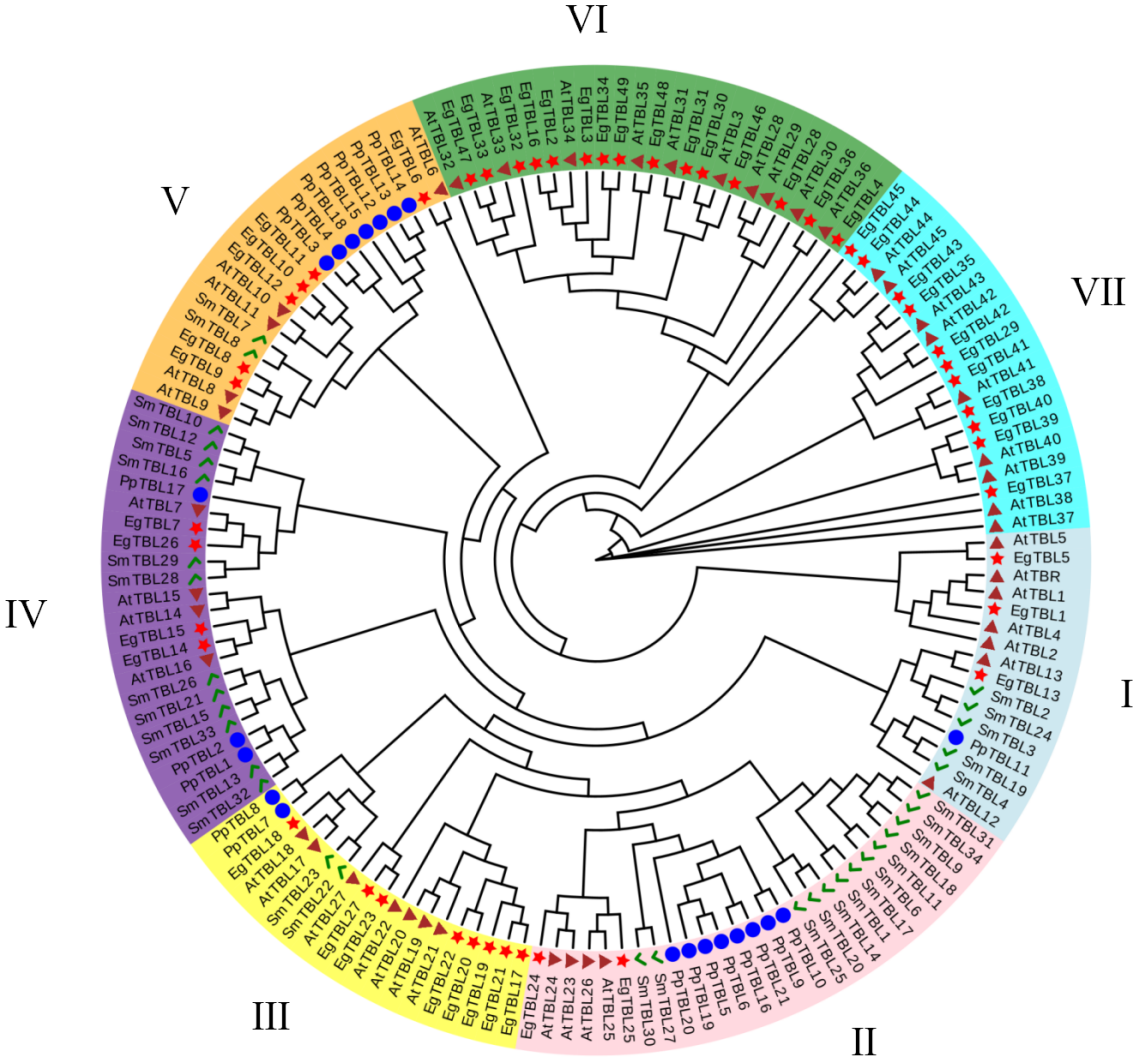
Comprehensive phylogenetic tree construction of TBL genes. AtTBL proteins are from *A. thaliana*; PpTBL proteins are from *P. patens*; EgTBL proteins are from *E. grandis*; SmTBL proteins are from *S. moellendorffii*.

**Figure 2:**
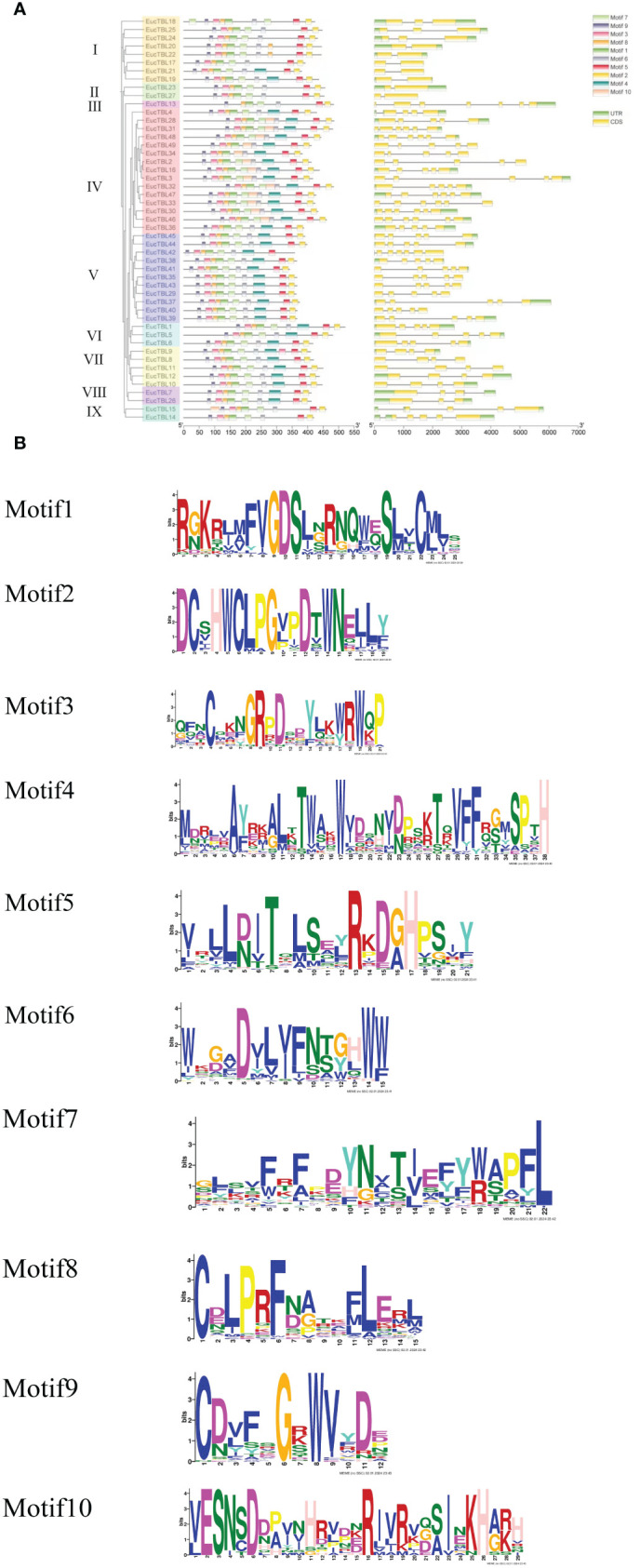
Structure of *E grandis* TBL gene and structure of expressed proteins. **(A)** The left side of the figure shows the clustering based on the phylogenetic tree. The middle of the figure shows the conserved motif structures indicated by different color blocks, and the right side of the figure shows the gene structures. **(B)** Sequence maps of different conserved motifs.

All TBL genes collected from *S. moellendorffii*, *P. patens*, *A. thaliana*, and *E. grandis* were divided into seven groups labeled I–VII (**Figure 1**). Interestingly, groups VI and VII only contained TBL genes from Arabidopsis and Eucalyptus (**Figure 2A**), while the TBL family members of *S. moellendorffii* and *P. patens* were significantly divergent at groups I–V. It was also found that TBL genes related to acetylation, including TBL28–35 in Arabidopsis, were mainly clustered in group VI (Qaseem and Wu, 2020). Similarly, Eucalyptus TBL family members were also divided into nine subfamilies and named I to IX (**Figure 2A**). It was also shown that the number of exons of the Eucalyptus TBL gene family varied from two to seven (**Figure 2A**).

### 3.3 Conserved protein motif analysis and localization of the TBL gene on chromosomes

A total of 10 conserved protein motifs, Motif 1–Motif 10 (**Figure 2B**), were screened in this study. The number of conserved motifs in TBL family members ranged from 8 to 10. From the phylogenetic tree, *TBL*s of group IV (except *EgTBL4* and *EgTBL36*) have 10 conserved motifs, and all of the other *TBL*s, except group IV, have no Motif 10. In addition, most members of group I (except *EgTBL19* and *EgTBL21*) and all members of groups II, III, and IX have no Motif 4. Other conserved motifs except Motif 4 and Motif 10 basically exist in all members, even orientational consistent. Among the motifs, the sequence of Motif 2 is the most conserved (**Figure 2B**), and the typical amino acid sequence is DCXHWCLPGXXDXWN.

**Figure 3:**
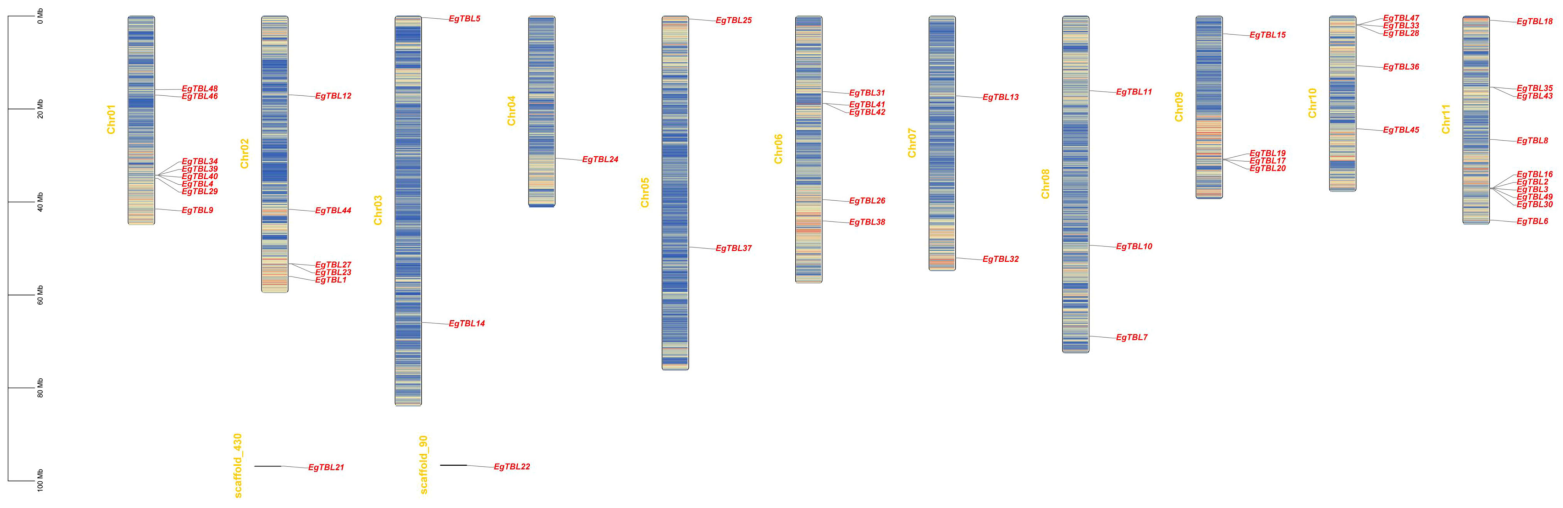
Localization map of *TBL* gene on chromosome. Baguettes are chromosomes, and short lines are sequences of unknown position not assembled to the chromosome; red font size markers are gene names; heatmap in the chromosome is gene content per 100,000-bp sequence.

A total of 47 TBL genes are uneven localized on 11 chromosomes, with 10 TBL family members distributed on chromosome 11 and only 1 member distributed on chromosome 4. Meanwhile, two TBL genes are localized on the scaffold. In addition, some TBL genes are clustered together on chromosomes as gene islands, especially on chromosome 11, with up to five genes localized in the same position (**Figure 3**).

### 3.4 TBL gene of *E. grandis* collinearity analysis

**Figure 4:**
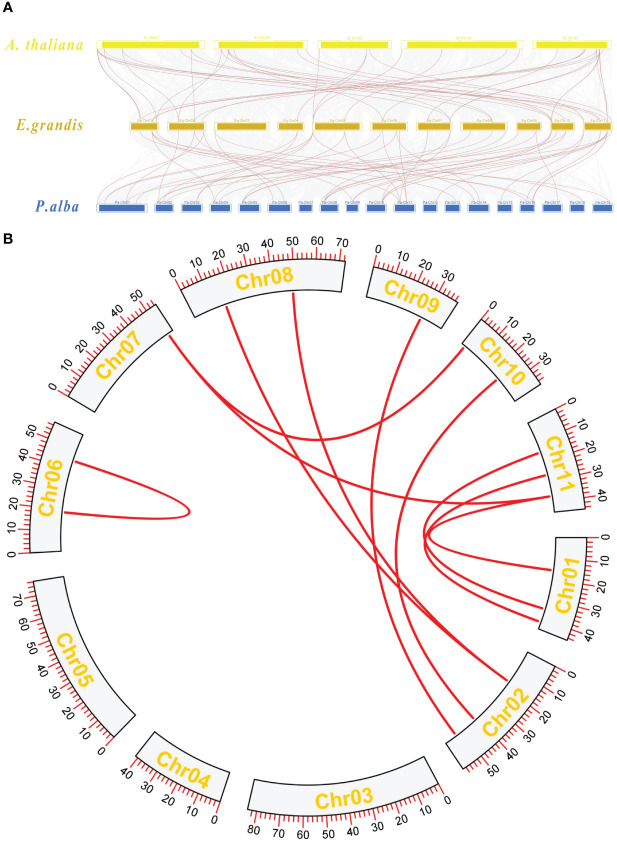
**(A)** Collinearity analysis plot. *A thaliana* chromosomes at the top, *E grandis* chromosomes in the center, and *P. alba* chromosomes at the bottom. **(B)** Intraspecific collinearity analysis of *Eucalyptus*.

Collinearity analysis among *A. thaliana*, *E. grandis*, and *P. alba* was performed. The TBL gene members were highlighted with red lines. The results showed that the *TBL* genes between *E. grandis* and *A. thaliana* had 44 gene pairs of collinearity (**Figure 4A**), while the TBL genes between *E. grandis* and *P. alba* had 53 pairs of collinearity (**Figure 4A**). Intraspecific collinearity analysis revealed the presence of 10 collinearity pairs within the species.

### 3.5 Analysis of cis-acting elements of the TBL gene of *E. grandis*

**Figure 5:**
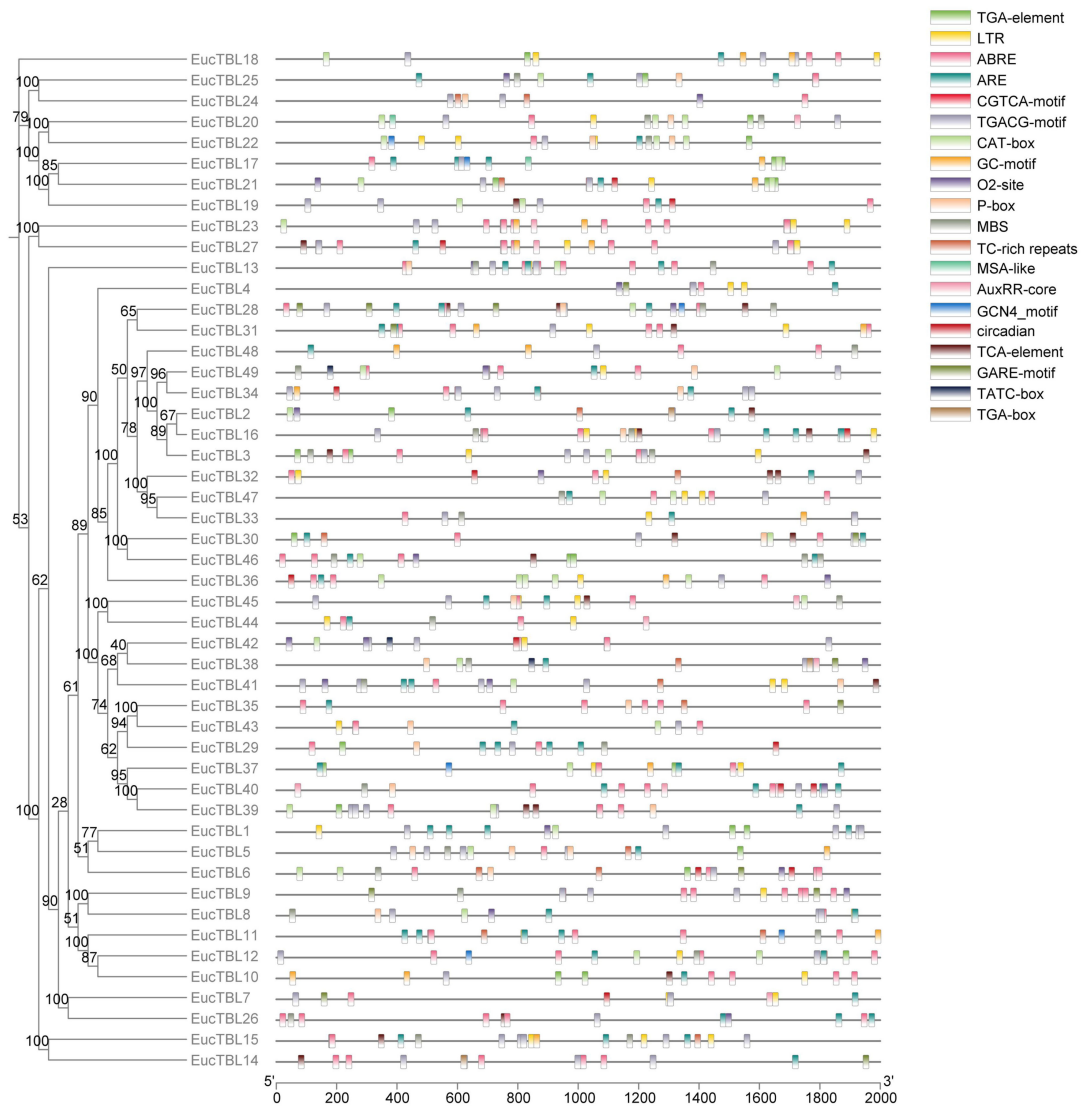
Cis-acting element prediction map. The horizontal axis of the figure is the 2,000-bp length upstream of the gene, the vertical axis of the figure is the gene ID, and the elements in the figure are detailed in the legend in the upper right corner.

According to promoter analysis, cis-acting elements were associated with the plant stress tolerance and hormone responses (**Figure 5**). The most widely distributed hormone-related cis-acting elements were ABRE, CGTCA-motif, and TGACG-motif. ABRE was the cis-acting element related to abscisic acid response, and the latter two were the cis-acting elements related to MeJA. The remaining widely distributed cis-acting elements are LTRs and TC-rich repeats, the former being hypothermia-related and the latter being stress-responsive. Other cis-acting elements and the physiological processes are shown in **Supplementary Table 1**.

### 3.6 Expression profiles of *TBL* genes among various tissues in *E. grandis*

**Figure 6:**
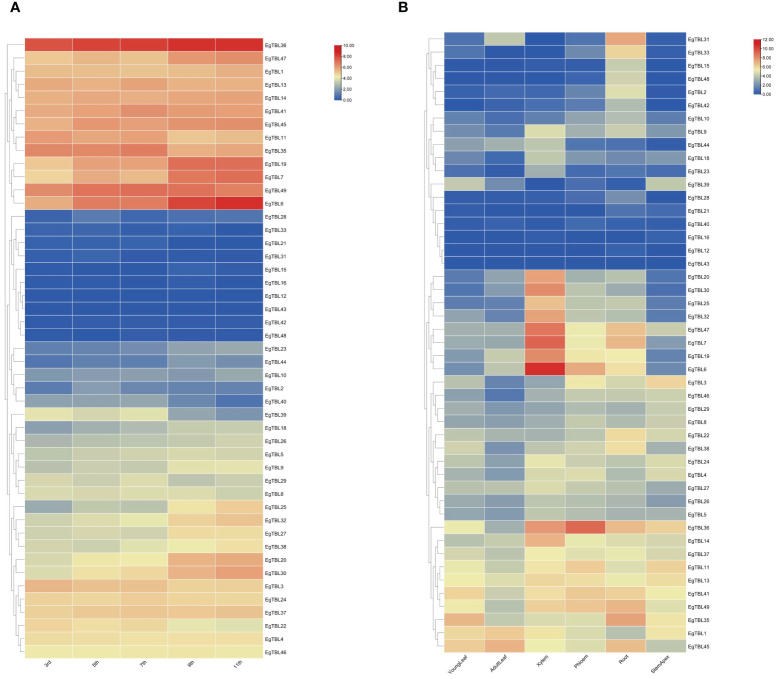
Expression profiles of TBL gene in various internodes of stem from the 3rd internode to the 11th internode **(A)** and in various tissues including adult leaves, young leaves, xylem, phloem, roots, and stem apex **(B)** in *E grandis*.

TBL gene expression in different internodes of 6-month-old *E. grandis* is shown in **Figure 6A**. From the top to the bottom of the tree, the expression of *EgTBL19*, *EgTBL20*, *EgTBL30*, *EgTBL36*, *EgTBL6*, and *EgTBL7* genes gradually increased, while the expression of *EgTBL11*, *EgTBL22*, *EgTBL3*, *EgTBL35*, and *EgTBL39* gradually decreased. **Figure 6B** shows the expression of TBL genes in different parts of *E. grandis* at half a year of age, and in general, the highest expression was found in the xylem of *E. grandis*, while the expression in leaves and terminal buds was smaller. As far as the combined expression profile of different genes was analyzed, *EgTBL6*, *EgTBL7*, *EgTBL36*, and *EgTBL47* had the highest expression. The most noteworthy gene was *EgTBL6*, whose expression in xylem was particularly prominent, nearly three times that of the next highest expressed gene.

### 3.7 Expression profiles of *TBL* genes’ response to various treatments in *E. grandis*

**Figure 7:**
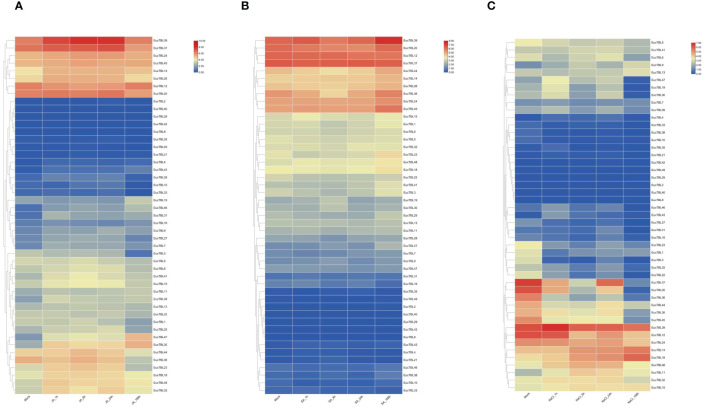
TBL gene expression at different times under jasmonic acid **(A)**, salicylic acid **(B)**, and salt stress treatment **(C)** in *E grandis*.

The transcriptome results of stress treatments at different times and sites showed that external stresses significantly affected the expression of *E. grandis* TBL gene family members. The expression of TBL genes in *E. grandis* seedling leaves varied with time under JA, SA, and salt stress treatments. Under JA treatment (**Figure 7A**), the expression of *EgTBL14*, *EgTBL18*, *EgTBL20*, *EgTBL23*, *EgTBL24*, *EgTBL26*, *EgTBL30*, *EgTBL32*, *EgTBL37*, *EgTBL39*, *EgTBL47*, and *EgTBL48* was not only higher but also significantly upregulated over time, while the expression of *EgTBL12*, *EgTBL3*, and *EgTBL36* were downregulated with time. Under SA treatment (**Figure 7B**), the expressions of *EgTBL20*, *EgTBL36*, *EgTBL44*, and *EgTBL3* were downregulated, whereas the expressions of no TBL gene were significantly upregulated. Under the salt stress treatments, the expression of *EgTBL1*, *EgTBL12*, *EgTBL20*, *EgTBL22*, *EgTBL23*, *EgTBL24*, *EgTBL26*, *EgTBL3*, *EgTBL36*, *EgTBL37*, *EgTBL44*, *EgTBL45*, and *EgTBL5* was downregulated, while the expression of *EgTBL11*, *EgTBL14*, *EgTBL18*, and *EgTBL48* was upregulated (**Figure 7C**). It is worth noting that the up- or downregulation of expression that we have illustrated is more of a short-term effect of treatment, and in terms of the negative feedback regulation that may be present in plants, it is normal for this short-term effect to be dialed back after a long period of treatment.

### 3.8 Expression profiles of *TBL* genes in adventitious root induced in *E. grandis*

**Figure 8:**
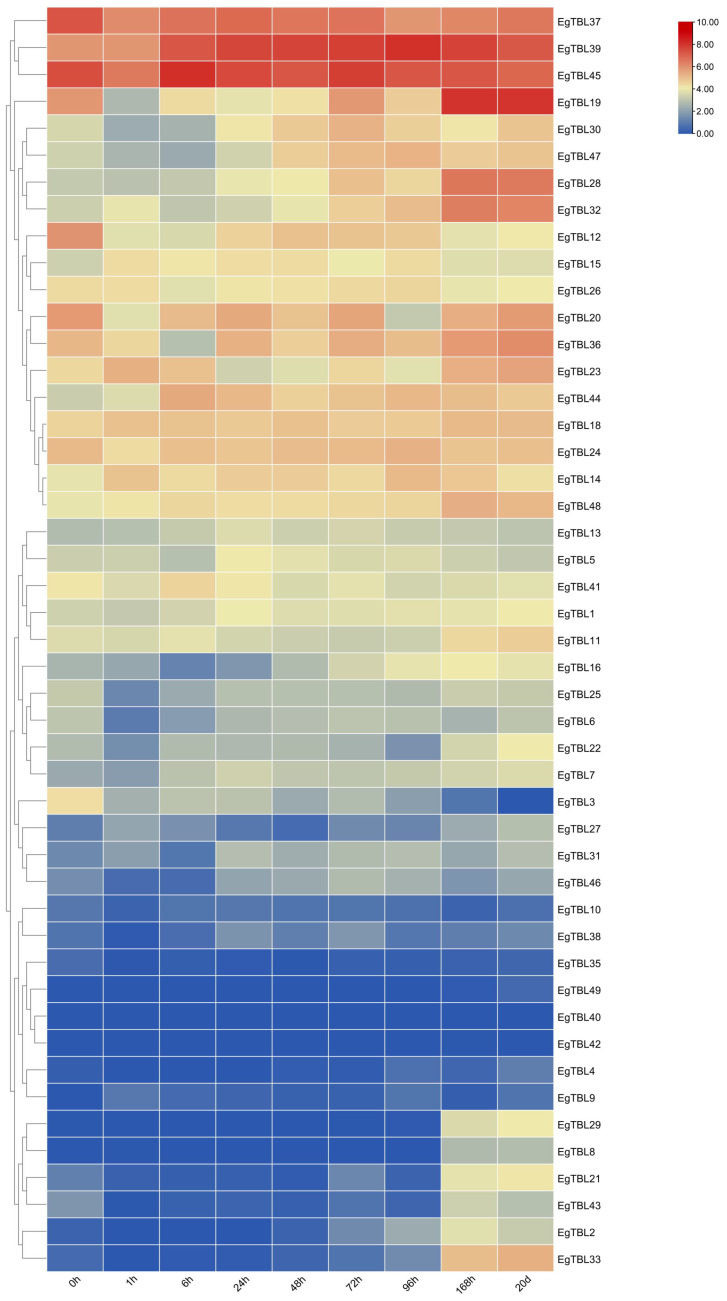
TBL gene expression in adventitious roots induced in tissue culture in *E. grandis*.

**Figure 8** shows the expression of TBL genes in adventitious roots induced at different growth stages when seedlings were grown in tissue culture. As *E. grandis* grew in tissue culture, the expression of *EgTBL12* and *EgTBL37* was downregulated and the expression of *EgTBL19*, *EgTBL23*, *EgTBL28*, *EgTBL32*, *EgTBL33*, *EgTBL39*, *EgTBL44*, *EgTBL47*, and *EgTBL48* was upregulated in the roots. *EgTBL19*, *EgTBL37*, *EgTBL39*, and *EgTBL45* had the highest expression overall at the base of the stem and in the roots during culture.

## 4 Discussion

*E. grandis* is an important timber species in the tropical and subtropical regions of the world, which meets the demand for wood and paper production in the development process of various countries. *TBL* genes are widely present in the genomes of higher plants, and studies have shown that they are essential for the acetylation of xylan in the plant cell wall, which is beneficial for the improvement of cell wall strength and timber properties, as well as improving the resilience of the trees. Thus, the study of the TBL gene family in Eucalyptus will contribute to forest improvement efforts.

The proteins of *E. grandis* TBL family members are hydrophilic, have a high instability index, and are mostly basic. Phylogenetic tree analysis reveals that the genome of the primitive eukaryote *C. reinhardtii* lacks TBL family members. In contrast, more complex organisms like *S. moellendorffii* and *P. patens* possess a few TBL gene family members, albeit distantly related to those in higher plants like *E. grandis* and *A. thaliana*. These results suggest that the TBL gene family evolved and expanded throughout plant evolution, playing a significant role in environmental adaptation. Overall, the protein sequences and structural domains within the *E. grandis* TBL family are highly conserved, indicating the gene’s pivotal role in plant biology and warranting further exploration of its functions. The study of conserved motifs (**Figure 2**) revealed that the unique Motif 10 structure in group IV members exhibits notable conservation. This conservation is instrumental in understanding TBL proteins’ functionality, particularly in distinguishing the functional nuances between group IV proteins and other TBL proteins, thereby aiding in a more thorough investigation and precise classification of the TBL family. The functional evolution and diversification of gene families often stem from gene fragment and tandem duplications, a phenomenon extensively observed in *A. thaliana* studies (Cannon et al., 2004; Levasseur and Pontarotti, 2011). The results of the intraspecific collinearity analysis further confirmed that the development of the TBL gene family in *E. grandis* is associated with this phenomenon. In group VII (**Figure 1**), the divergent branching of *Arabidopsis* and *E. grandi*s TBL genes likely results from fragment duplication. Chromosomal localization data reveal a tendency for gene clustering within the *E. grandis* TBL family, suggesting segmental duplication leading to tandem repeats (De Smet et al., 2002; **Figure 4B**). Collinearity analysis further corroborates this, showing increased collinearity pairs among higher plants and significant homology, thereby underscoring the gene’s conservation.

Analysis of *E. grandis* seedlings’ expression data under various stress treatments (**Figure 7**) emphasizes the significance of jasmonic acid (JA), a crucial phytohormone. JA plays a pivotal role in maintaining ion homeostasis, as evidenced in maize seedling studies (Mir et al., 2018), and is instrumental in the cellular stress response (Piotrowska et al., 2009; Xia et al., 2018; Shri et al., 2019). qRT-PCR studies have demonstrated JA’s role in enhancing tomato plants’ resistance to the heavy metal salt Pb via diverse pathways and in producing heat shock proteins within plants (Kochian et al., 2015; Bali et al., 2019; Nadarajah, 2020). In *E. grandis* seedlings treated with JA, there was a general upregulation in *TBL* gene expression. This, coupled with cis-acting progenitor analysis results, strongly suggests a close regulatory relationship between this gene family and JA. In *E. grandis* seedlings, SA treatment notably upregulated the expression of the *EgTBL37* gene in leaves. SA is known to be vital for developing systemic acquired resistance in tobacco and mitigating drought stress effects in winter wheat (Gaffney et al., 1993; Khalvandi et al., 2021). Therefore, the upregulated *EgTBL37* gene post-SA treatment is likely crucial in plant resistance processes. Abiotic stress significantly influences forest tree breeding (Harfouche et al., 2014), with gene expression under salt stress serving as a critical aspect of forest tree genetic improvement. Thus, researching relevant *TBL* genes is imperative. The TBL gene’s expression predominantly decreased under salt stress, indicating its potential adverse effect on the plant’s salt stress resistance.

Analysis of the TBL gene family expression in adventitious roots of group-cultivated *E. grandis* seedlings over various time periods (**Figure 8**) reveals a general upregulation correlating with the development of adventitious roots. This trend underscores the *TBL* genes’ significant role in promoting adventitious rootogenesis. Expression data from various parts of 6-month-old *E. grandis* (**Figure 6B**) highlight a pronounced expression of *TBLs* in the xylem, with *EgTBL6* being especially prominent. Analysis of different internodes (**Figure 6A**) shows increasing *TBL* gene expression and lignification towards the tree base. These findings further establish the crucial role of *TBL* genes in the lignification process of *E. grandis*.

**Figure 9:**
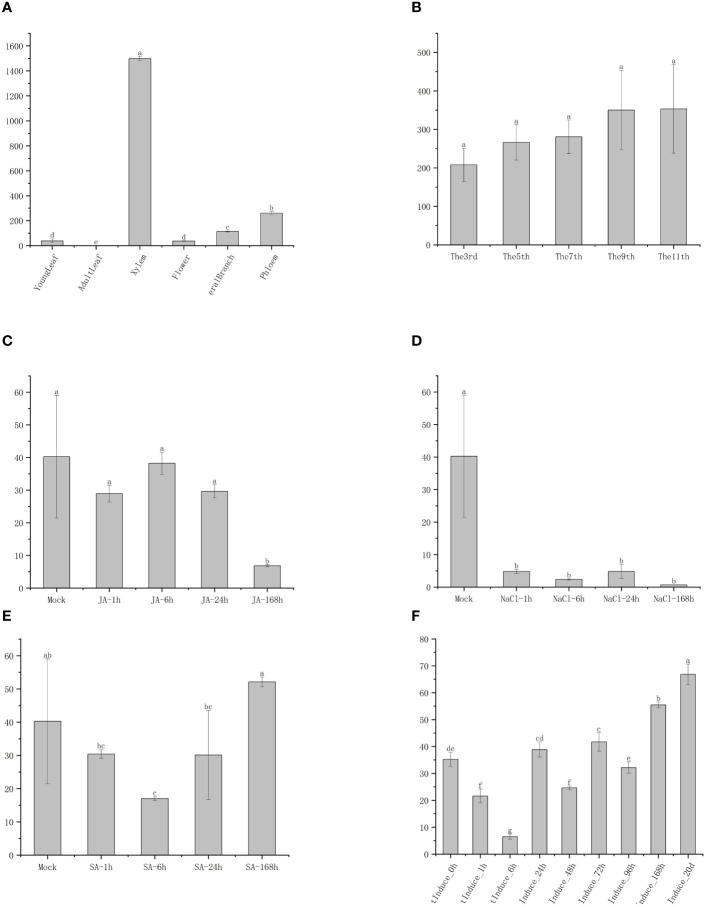
**(A)** Graph of the expression of the gene in different parts of eucalyptus in half a year. **(B)** Graph of the difference of the expression of the gene among different knots of eucalyptus. **(C)** Graph of the change of the expression of the gene in eucalyptus under salicylic acid treatment. **(D)** Graph of the change of the expression of the gene in eucalyptus under salt stress. **(E)** Graph of the change of the expression of the gene in eucalyptus under jasmonic acid treatment. **(F)** Graph of the change of the expression of the gene in eucalyptus in the root at different times in the process of tissue culture.

The TBL family is prominently known for its O-acetyl polysaccharide transferase activity in cell wall acetylation (Yuan et al., 2016a; Zhong et al., 2017; Stranne et al., 2018), with xylan acetylation playing a vital role in augmenting plants’ stress tolerance (Vogel et al., 2004; Pogorelko et al., 2013; Jiang et al., 2014; Gao et al., 2018). The *TBL28–TBL35* genes, crucial in the xylan acetylation process, were highlighted in Arabidopsis studies (Yuan et al., 2016b; Yuan et al., 2016c). In the phylogenetic tree encompassing various species, these genes belong to group VI (group IV in *E. grandis*), specifically *EgTBL2* and *EgTBL3*, among others. Notably, group IV *E. grandis TBL* genes are characterized by Motif 10 with a conserved ESNXD sequence and additionally have a conserved arginine and a proline at positions 16 and 26, respectively. From the results of chromosomal localization, the gene members of group IV were not highly concentrated on individual chromosomes and were generally dispersed. The group IV Eucalyptus *TBL* gene that was significantly upregulated under JA treatment and downregulated under salt stress was *EgTBL36*, demonstrating that it may play an important role in response to stress (**Figures 7**, **9**). The results of the significance analysis of the expression of this gene showed that the gene was prominently expressed in the xylem of *E. grandis* and that there was a significant short-term downregulation of its expression under salt stress, SA, and JA treatments, and these results reinforced the conjecture that it plays an important function in the resistance and development of *E. grandis*. From the expression data of different internodes of 6-month-old *E. grandis*, the expression of *EgTBL36* gene was even more fault leading (**Figure 6**). These results suggest that *EgTBL36* gene may be a key functional gene for xylan acetylation in *E. grandis* (**Figure 9**).

## 5 Conclusion

We analyzed the TBL gene of *E. grandis* in terms of phylogeny, gene structure, and proteome. While 49 gene members of the TBL family were identified, they were divided into nine subfamilies. A number of conserved protein structures were identified, and phylogenetic and kinship analyses among different species further ensured the accuracy of the family identification. The expression profiles of different treatments and sites revealed that the *TBL*s of *E. grandis* play an important role in the plant’s resistance to environmental stresses, and the process is related to its function of xylan acetylation. Based on the distribution of the family members that play important roles in *A. thaliana* and referring to the expression profiling data, we identified the key genes that may play roles in the TBL family of *E. grandis*, which will be helpful for the subsequent functional studies and will help breeders to carry out breeding for timber property improvement.

## Data availability statement

The datasets presented in this study can be found in online repositories. The names of the repository/repositories and accession number(s) can be found in the article/**Supplementary Material**.

## Author contributions

JT: Conceptualization, Data curation, Formal analysis, Funding acquisition, Investigation, Methodology, Project administration, Resources, Software, Supervision, Validation, Visualization, Writing – original draft, Writing – review & editing. TL: Data curation, Formal analysis, Investigation, Methodology, Writing – review & editing. HL: Data curation, Funding acquisition, Writing – review & editing. CF: Data curation, Investigation, Methodology, Writing – review & editing.

## Supplementary material

Supplementary Table 1 Table describing promoters and their functions.
