# Supplementary material for: Genome-wide analysis and identification of the TBL gene family in Eucalyptus grandis
Source: Front Plant Sci. 2024 Aug 7;15:1401298. doi: 10.3389/fpls.2024.1401298 (PMC11337025; doi:10.3389/fpls.2024.1401298)
Supplement: Supplementary Table 1 — Table describing promoters and their functions. [file DataSheet_1.docx]

Supplementary Table 1: Table describing promoters and their functions.

| **Promoter** | **Function**[40-42] |
| --- | --- |
| TGA-element | auxin-responsive element |
| MSA-like | cis-acting element involved in cell cycle regulation |
| TC-rich repeats | cis-acting element involved in defense and stress responsiveness |
| TATC-box | cis-acting element involved in gibberellin-responsiveness |
| LTR | cis-acting element involved in low-temperature responsiveness |
| TCA-element | cis-acting element involved in salicylic acid responsiveness |
| ABRE | cis-acting element involved in the abscisic acid responsiveness |
| ARE | cis-acting regulatory element essential for the anaerobic induction |
| AuxRR-core | cis-acting regulatory element involved in auxin responsiveness |
| circadian | cis-acting regulatory element involved in circadian control |
| CGTCA-motif | cis-acting regulatory element involved in the MeJA-responsiveness |
| TGACG-motif | cis-acting regulatory element involved in the MeJA-responsiveness |
| O2-site | cis-acting regulatory element involved in zein metabolism regulation |
| CAT-box | cis-acting regulatory element related to meristem expression |
| GCN4_motif | cis-regulatory element involved in endosperm expression |
| GC-motif | enhancer-like element involved in anoxic specific inducibility |
| GARE-motif | gibberellin-responsive element |
| P-box | gibberellin-responsive element |
| MBS | MYB binding site involved in drought-inducibility |
| TGA-box | part of an auxin-responsive element |
